# Parathyroid hormone (1-34) promotes the effects of 3D printed scaffold-seeded bone marrow mesenchymal stem cells on meniscus regeneration

**DOI:** 10.1186/s13287-020-01845-x

**Published:** 2020-07-30

**Authors:** Wen Zhao, Tong Zou, Hao Cui, Yangou Lv, Dengke Gao, Chenmei Ruan, Xia Zhang, Yihua Zhang

**Affiliations:** College of Veterinary Medicine, Northwest A&F University, Yangling, 712100 Shaanxi China

**Keywords:** Tissue engineering, Meniscus, BMSCs, PTH (1-34), Articular cartilage

## Abstract

**Background:**

Cell-based tissue engineering represents a promising management for meniscus repair and regeneration. The present study aimed to investigate whether the injection of parathyroid hormone (PTH) (1-34) could promote the regeneration and chondroprotection of 3D printed scaffold seeded with bone marrow mesenchymal stem cells (BMSCs) in a canine total meniscal meniscectomy model.

**Methods:**

3D printed poly(e-caprolactone) scaffold seeded with BMSCs was cultured in vitro, and the effects of in vitro culture time on cell growth and matrix synthesis of the BMSCs–scaffold construct were evaluated by microscopic observation and cartilage matrix content detection at 7, 14, 21, and 28 days. After that, the tissue-engineered meniscus based on BMSCs–scaffold cultured for the appropriate culture time was selected for in vivo implantation. Sixteen dogs were randomly divided into four groups: PTH + BMSCs–scaffold, BMSCs–scaffold, total meniscectomy, and sham operation. The regeneration of the implanted tissue and the degeneration of articular cartilage were assessed by gross, histological, and immunohistochemical analysis at 12 weeks postoperatively.

**Results:**

In vitro study showed that the glycosaminoglycan (GAG)/DNA ratio and the expression of collagen type II (Col2) were significantly higher on day 21 as compared to the other time points. In vivo study showed that, compared with the BMSCs–scaffold group, the PTH + BMSCs–scaffold group showed better regeneration of the implanted tissue and greater similarity to native meniscus concerning gross appearance, cell composition, and cartilage extracellular matrix deposition. This group also showed less expression of terminal differentiation markers of BMSC chondrogenesis as well as lower cartilage degeneration with less damage on the knee cartilage surface, higher expression of Col2, and lower expression of degeneration markers.

**Conclusions:**

Our results demonstrated that PTH (1-34) promotes the regenerative and chondroprotective effects of the BMSCs–3D printed meniscal scaffold in a canine model, and thus, their combination could be a promising strategy for meniscus tissue engineering.

## Background

Meniscus injury severely limits knee function and increases the risk of osteoarthritis [1]. In recent years, the rapid development in the fields of tissue engineering and regenerative medicine has provided a promising treatment for meniscus injury [2, 3]. The main steps involved in meniscus tissue engineering are preparing a scaffold and seeding cells and regulating the cell–scaffold construct through cytokines, mechanical stimulation, and other methods to synthesize the extracellular matrix (ECM) in vitro, followed by its transplantation in vivo for meniscus regeneration and function [4]. 3D printing technology can fabricate scaffolds with complete control of size, shape, and porosity; it has been used in many previous studies to prepare tissue-engineered meniscus scaffolds [5–8]. Bone marrow mesenchymal stem cells (BMSCs) are easy to isolate and proliferate, have low immunogenicity, and have the potential to differentiate into cartilage; thus, they have become ideal seed cell for meniscus tissue engineering [9, 10]. However, the available literature does not answer several problems related to their use.

First, the accumulation of a certain amount of ECM in the in vitro culture is conducive to the better functioning of the cell–scaffold construct in response to knee pressure [11]. However, the influence of the in vitro culture time on the growth and differentiation of seed cells in meniscus tissue engineering has not been determined. Therefore, in order to select the appropriate implantation time, it is necessary to determine the effects of in vitro culture time on the cell growth and ECM accumulation on cell–scaffold constructs. Second, BMSCs often undergo terminal differentiation during chondrogenesis, which hampers the regenerative efficacy of tissue engineering [12, 13]; this limitation needs to be addressed when using BMSCs as seed cells for meniscus tissue engineering. Furthermore, although tissue-engineered meniscus transplantation can promote the recovery of knee joint function, it also causes different degrees of damage to knee cartilage and subchondral bone [14, 15].

Parathyroid hormone (PTH) (1-34) inhibits the hypertrophy of BMSCs during chondrogenic differentiation [16, 17], inhibits articular cartilage degeneration, and promotes articular chondrocyte proliferation and ECM synthesis [18]. Therefore, it is often used in studies on cartilage tissue engineering as well as in osteoarthritis treatment [19–21]. Orth et al. [22] reported that injection of PTH (1-34) into a rabbit model of osteochondral defect significantly increased the expression of PTH receptor in chondrocytes and osteocytes in the rabbit joint, improved the surface structure and integration of articular cartilage, and reconstructed the subchondral bone. Zhang et al. [23] combined parathyroid hormone-related protein (PTHrP) treatment with collagen–silk scaffold implantation and found that it improved the osteochondral defect repair efficacy. However, to the best of our knowledge, there are currently no reports on the repair of meniscus defects by combining PTH (1-34) and tissue-engineered meniscus.

In this study, we aimed to determine the appropriate in vitro culture time for 3D printed meniscus scaffolds seeded with canine BMSCs. Further, we transplanted these into meniscectomy model dogs to investigate the repair potential of PTH (1-34) combined with 3D printed scaffold–BMSCs on meniscus defect. Thus, this study may provide a reference approach for meniscus tissue engineering in large animals and humans.

## Methods

### Fabrication of 3D printed scaffolds

A native medial meniscus of the right knee was acquired from a skeletally mature dog after approval from the Institutional Animal Care and Use Committee of Northwest A&F University. The meniscus was laser scanned (Handy SCAN 700, Creaform, Canada), and the data were imported into process software (Vxelements, Creaform, Canada) to reconstruct a three-dimensional (3D) model. The print parameters of a 3D bioprinter (BIOPLATFORM, Medprint, China) were adjusted (Table 1) as described in a previous study [6], and a poly(e-caprolactone) (PCL) (Changchun SinoBiomaterials, China) wire (43–50 kDa) was melted and extruded through a heated metal nozzle to print the scaffold. Simultaneously, the same printing parameters were used to print cylindrical scaffolds, with 5-mm diameter and 3-mm thickness, for the cell compatibility tests of the scaffold.

**Table 1:** Print parameters of the meniscus scaffold

Print property	Parameters
PCL wire diameter	1.75 mm
Temperature set point	90 °C
Fiber diameter	0.3 mm
Layer thickness	0.3 mm
Wall thickness	0.8 mm
Porosity	60%
Pore interconnectivity Mass ($\overline{\mathrm{x}}$ ± SD, *n* = 10)	100% 0.0894 ± 0.0084 g

### Isolation and culture of BMSCs

BMSCs were isolated from canine new stillbirths’ (fetus that died during delivery) bone marrows and identified as reported in previous work [24]. BMSC culture medium consisted of Minimum Essential Medium alpha (α-MEM, Gibco, Billings, MT, USA) supplemented with 10% fetal bovine serum (FBS; Sigma, USA) and 1% penicillin–streptomycin (Sigma).

### Scaffold characterization

#### Microstructure

The microstructure and pore size of scaffolds were observed and measured by scanning electron microscopy (Nova SEM-450, FEI, USA) after freeze-drying and coating with a 5-nm layer of gold on the surface.

#### Porosity

The weight of the dry PCL scaffold was recorded as Ws; a pycnometer filled with ethanol was weighted and recorded as W1. Put the scaffold into the pycnometer and then vacuum to extract the air out of the scaffold. Filled up the pycnometer with ethanol again and took the weight (W2). After then, took out the scaffold and get the weight of the rest ethanol and the pycnometer (W3). The porosity was calculated according to the formula: porosity (%) = (W2 − W3 − Ws)/(W1 − W3).

#### Degradation rate

The weight of the dry PCL scaffold was recorded as W1, and it was then soaked in phosphate-buffered saline (PBS) at 37 °C and pH 7.4. After 4, 8, and 12 weeks, three scaffolds were removed and dried at 45 °C for 24 h each time, and the weight was recorded as W2. The in vitro degradation rate was calculated according to the formula: degradation rate (%) = (W1 − W2)/W1 × 100.

#### Cytocompatibility

CCK-8 cytotoxicity test was used to evaluate the compatibility of PCL scaffolds.

Fourth passage BMSCs were seeded in a 96-well plate at a density of 3 × 10^3^/well in 100 μL of BMSC culture medium and incubated at 37 °C with 5% CO_2_ for 4 h, then the cylindrical PCL scaffold was placed gently into the plate and cocultured with BMSCs. BMSCs cultured in monolayer assessed as the control group. At days 3, 5, and 7 of culture, 10 μL of CCK-8 solution (ZETA life, USA) was added to each well of the plate and incubated for 4 h at 37 °C. Thereafter, 100 μL of the solutions was transferred to another standard 96-well plate, and the optical density (OD) at 450 nm was measured using a microplate reader (Tecan, Switzerland).

### Preparation of tissue-engineered meniscus

#### Cell seeding and in vitro culture of scaffold

The 3D printed PCL scaffolds were sterilized by UV irradiation and soaked in α-MEM. The fourth passage BMSCs were resuspended to 6 × 10^6^/mL, 500 μL of the cell suspension was dripped on the upper surface of the scaffold, and the scaffold was placed in an incubator (37 °C, 5% CO_2_) for 3 h to aid cell attachment; it was then turned over and the procedure was repeated two times. The BMSCs–scaffold construct was then cultured in chondrogenic induction medium consisting of high-glucose Dulbecco’s modified Eagle’s medium (DMEM; Gibco), 1% penicillin–streptomycin, 40 ng/mL dexamethasone, 50 μg/mL l-proline, 50 μg/mL ascorbate 2-phosphate, 1 mmol/L insulin–transferrin–selenium (ITS), 1 mmol/L sodium pyruvate (all Sigma), 10 ng/mL transforming growth factor-β3 (TGF -β3; Peprotech, USA), and 10 ng/mL bone morphogenetic protein-2 (BMP-2; Peprotech). Cell growth of BMSCs–scaffold construct was observed by inverted phase-contrast microscopy.

#### Assessment of ECM accumulation at different differentiation culture time points

BMSCs–scaffold constructs cultured for 7, 14, 21, and 28 days were digested in 125 μg/mL papain solution (Sigma) at 55 °C overnight and then centrifuged at 10,000*g* for 10 min; the supernatant was collected for glycosaminoglycan (GAG) and DNA determination. GAG quantification was performed using the Blyscan Glycosaminoglycan Assay (Biocolor, UK); briefly, specimens were complexed with Blyscan dye, the absorbance was measured at 656 nm, and the concentration was calculated using a standard curve. DNA content was determined with PicoGreen kit (Invitrogen, USA); the sample and dsDNA standard solution were incubated with the Picogreen dye, and the fluorescence value was detected. Ex/Em = 480 nm/520 nm, the DNA concentration of the sample (ng/mL) was calculated using the standard curve. The GAG/DNA ratio was used to evaluate the accumulation of GAG.

Western blotting was used to determine the collagen type II (Col2) expression in the samples. BMSCs–scaffold constructs were treated by western blotting procedure with the labeling of Col2 (1,1000; Abcam, UK), and immunoblots were visualized by chemiluminescence using an HRP substrate (Millipore, USA). PCNA was used as a loading control.

### Animal model

All animal procedures were approved by the Institutional Animal Care and Use Committee of Northwest A&F University. Sixteen mongrel dogs, aged 2–5 years and weighing 7 ± 1 kg, were randomly divided into 4 groups: PTH + BMSCs–scaffold group, BMSCs–scaffold group, Sham group, and Meniscectomy group, with 4 dogs in each group.

**Fig. 1:**
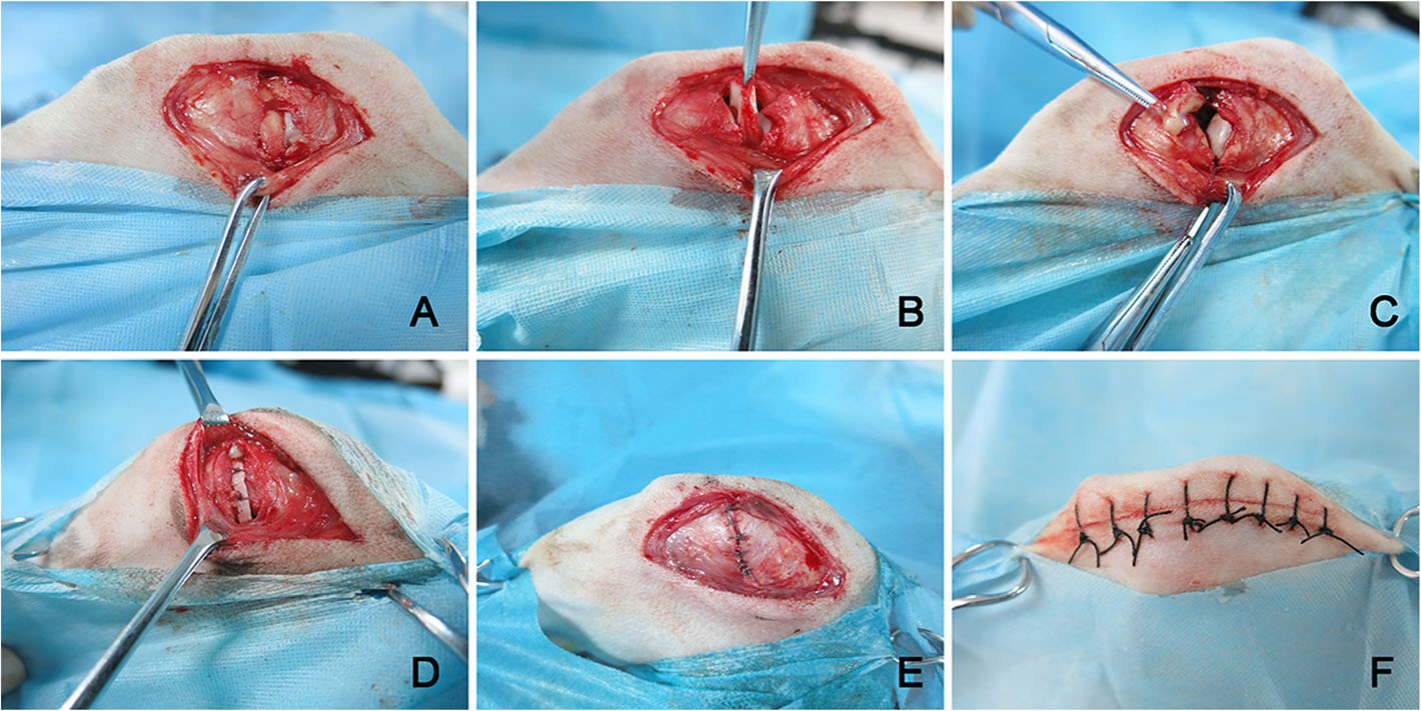
Implantation process of the tissue-engineered meniscus. **a** Cutting the skin, fascia, and joint capsule. **b** Separating medial meniscus. **c** Resection of the medial meniscus. **d** Transplanted tissue-engineered and sutured meniscal implant. **e** Suture joint capsule. **f** Sutured skin incision

After surgery preparations and anesthetizing the animals, a medial parapatellar approach [25] (Fig. 1) was used on the right knee of the animal to expose the medial meniscus. The capsula articularis was cut laterally along the proximal edge of the medial meniscus, and the entire medial meniscus was removed. For the PTH + BMSCs–scaffold group and BMSCs–scaffold group, the tissue-engineered scaffold was placed in the anatomically correct position and then sutured to the anterior and posterior ligaments and the adjacent synovium using 4-0 Polyglycolic Acid suture (Ethicon, Johnson & Johnson Medical B.V.); the joint capsule, subcutaneous tissue, and skin were closed gradually with 3-0 suture (Ethicon). For the Meniscectomy group, only total resection of the meniscus was performed, while for the Sham group, the sham operation was performed involving exposure of the meniscus followed by closure in layers. The operation sites were isolated with sterilized gauze and splinted for external fixation. Postoperative analgesia and antibiotic prophylaxis were performed for 5 days. The splint was removed 7 days postoperatively, and the animals were taken for regular walks 2 weeks postoperatively to promote knee rehabilitation.

One week postoperatively, animals in the PTH combined group were intra-articularly injected on the right knee joint with2.4 μg/kg PTH (1-34) (CHINESE PEPTIDE, China) every 2 days for 3 weeks, while the animals in the other groups were injected with the same dose of normal saline.

### Postoperative observation and knee function score

After the operation, the health and rehabilitation conditions of the animals were observed and recorded. Twelve weeks postoperatively, the knee joint function was evaluated by three observers blinded to the groups based on limp, swelling, stair climbing, squatting, and locking, using a modified Lysholm score [25].

### Anatomic observation

At week 12 after the operation, the animals were euthanized and their knee joints were harvested. The menisci, femur, and tibia cartilages were observed and photographed; cartilages were blindly evaluated according to the International Cartilage Repair Society (ICRS) cartilage lesion classification [26] to assess the chondroprotective effects of implants and PTH.

### Histological evaluation

The implant specimens were fixed in 4% paraformaldehyde and embedded in paraffin, then sectioned into 5-μm thickness, and stained with hematoxylin and eosin (H&E) for general observation, toluidine blue (TB) for the presence of proteoglycans, and picrosirius red (PR) for the presence of collagen type I (Col1). The specimens of the femoral condyle and the tibial plateaus were fixed in 4% paraformaldehyde and decalcified in 10% ethylenediaminetetraacetic acid for 3 weeks. They were then embedded in paraffin, sectioned into 7-μm thickness, and stained with H&E for general observation and were blindly graded according to the modified Mankin score [27] to evaluate the damages of joint cartilage. The GAG of the tibial plateaus was stained with Safranin-O/Fast Green (SO/FG), and integrated optical density (IOD) value and area of the positive regions (red-stained) of each magnified image were measured using ImageJ 1.58 software (National Institutes of Health, USA); for semiquantitative analyses, the data were expressed as the average optical density (IOD/area).

### Immunohistochemistry

Immunohistochemical analyses were used to evaluate the expression level of the BMSC chondrogenesis terminal differentiation markers collagen type X (Col10), Col1, and matrix metalloproteinases-13 (MMP13) in the implants, as well as the expression of Col2, cartilage degradation markers MMP13, and A disintegrin and metalloproteinase with thrombospondin motifs 5 (Adamts5) in the tibial plateau cartilage. The antibodies for these proteins were purchased from Bioss, Beijing, China. The DAB substrate system (Zsbio, China) was used for color development. IOD value and area of positive regions of each magnified image were measured with ImageJ 1.58 software. For semiquantitative analyses, the data were expressed as the average optical density (IOD/area).

### Statistical analysis

All statistical data were expressed as mean ± standard deviation. SPSS 22.0 statistical software (IBM, USA) was used for statistical analysis. LSD-t test was performed for comparisons of means between two groups, and one-way analysis of variance (ANOVA) was used for comparisons among multiple groups. For all tests, *P* < 0.05 was considered statistically significant.

## Results

### Characterization of 3D printed scaffold

#### Structure

**Fig. 2:**
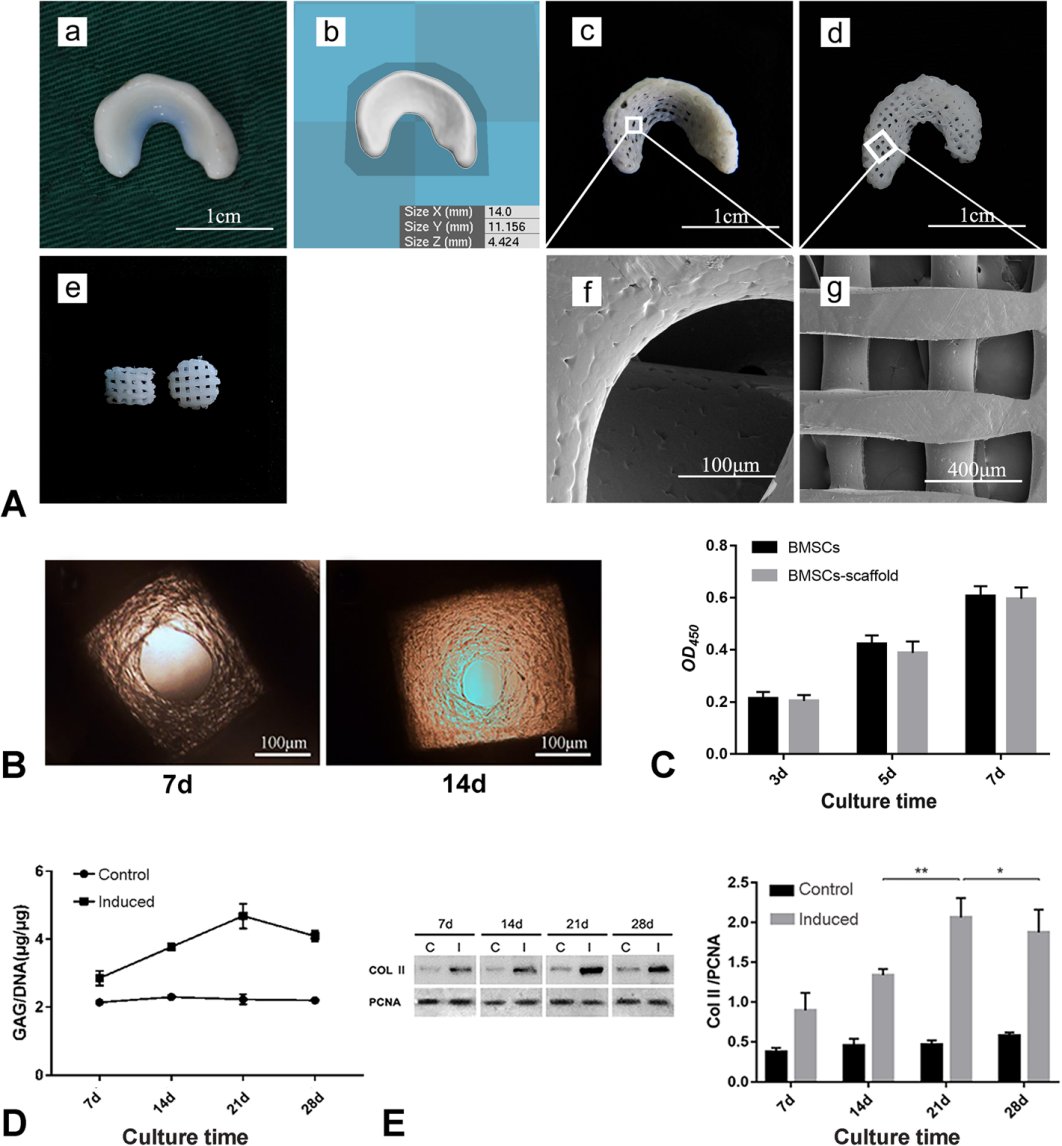
Preparation of tissue-engineered meniscus. **a** Characteristics of 3D printed scaffold: (a) canine native meniscus, (b) reconstruction model of canine native meniscus, (c) general observation of the upper surface of the 3D printed meniscus scaffold, (d) general observation of the bottom of the 3D printed meniscus scaffold, (e) cylindrical scaffolds with the same printing parameters as the 3D printed meniscus scaffold, and (f, g) scanning electron microscope images of the upper surface and bottom of the 3D printed meniscus scaffold. **b** Inverted phase contrast microscope image of BMSCs–scaffold constructs cultured for 7 days and 14 days. **c** CCK-8 assay for the determination of the cell compatibility of the 3D printed PCL scaffold. **d** GAG/DNA ratios of BMSCs–scaffold constructs at different culture time points. **e** The relative expression levels of Col2 in BMSCs–scaffold constructs at different culture time points (*P < 0.05, **P < 0.01)

The 3D printed meniscus scaffolds reproduced the native meniscus 3D geometry. SEM images showed that the pores in the scaffold were uniformly distributed and had a high degree of interconnectivity (Fig. 2A), and the pore size was 214.43 ± 13.5 μm.

#### Porosity

The measured porosity of the scaffold was consistent with the printing parameters, which was 61.19% ± 1.06%.

#### Degradation rate

The scaffolds showed relatively steady degradation in 12 weeks; the in vitro degradation rates at weeks 4, 8, and 12 were 0.78% ± 0.11%, 1.13% ± 0.05%, and 1.48% ± 0.13%, respectively.

#### Cell compatibility

In CCK-8 assay, the BMSCs showed an increased proliferative tendency during the culturing period (Fig. 2C); the OD values of the samples at 3, 5, and 7 days after seeding were 0.204 ± 0.023, 0.388 ± 0.044, and 0.596 ± 0.606, respectively, and those of the control group were 0.214 ± 0.025, 0.422 ± 0.033, and 0.616 ± 0.032, respectively. There was no significant difference between the two groups at each time point.

### ECM accumulation of BMSCs–scaffold constructs at different culture time points in vitro

The microscopy images (Fig. 2B) showed that BMSCs proliferated rapidly on the scaffold. The GAG/DNA ratio of the BMSCs–scaffold constructs increased with culture time and reached its peak at day 21, which was about twice the value at day 7, and then decreased at day 28 (Fig. 2D). In line with GAG/DNA determination, the expression of Col2 in the BMSCs–scaffold constructs increased continually until day 21 of culture; the gray value analysis showed that the expression on day 21 was significantly higher than that on day 14 and day 28 (*P* < 0.05, Fig. 2E). Therefore, the BMSCs–scaffold cultured for 21 days in vitro was selected for in vivo implantation.

### Postoperative observations and knee function score

**Fig. 3:**
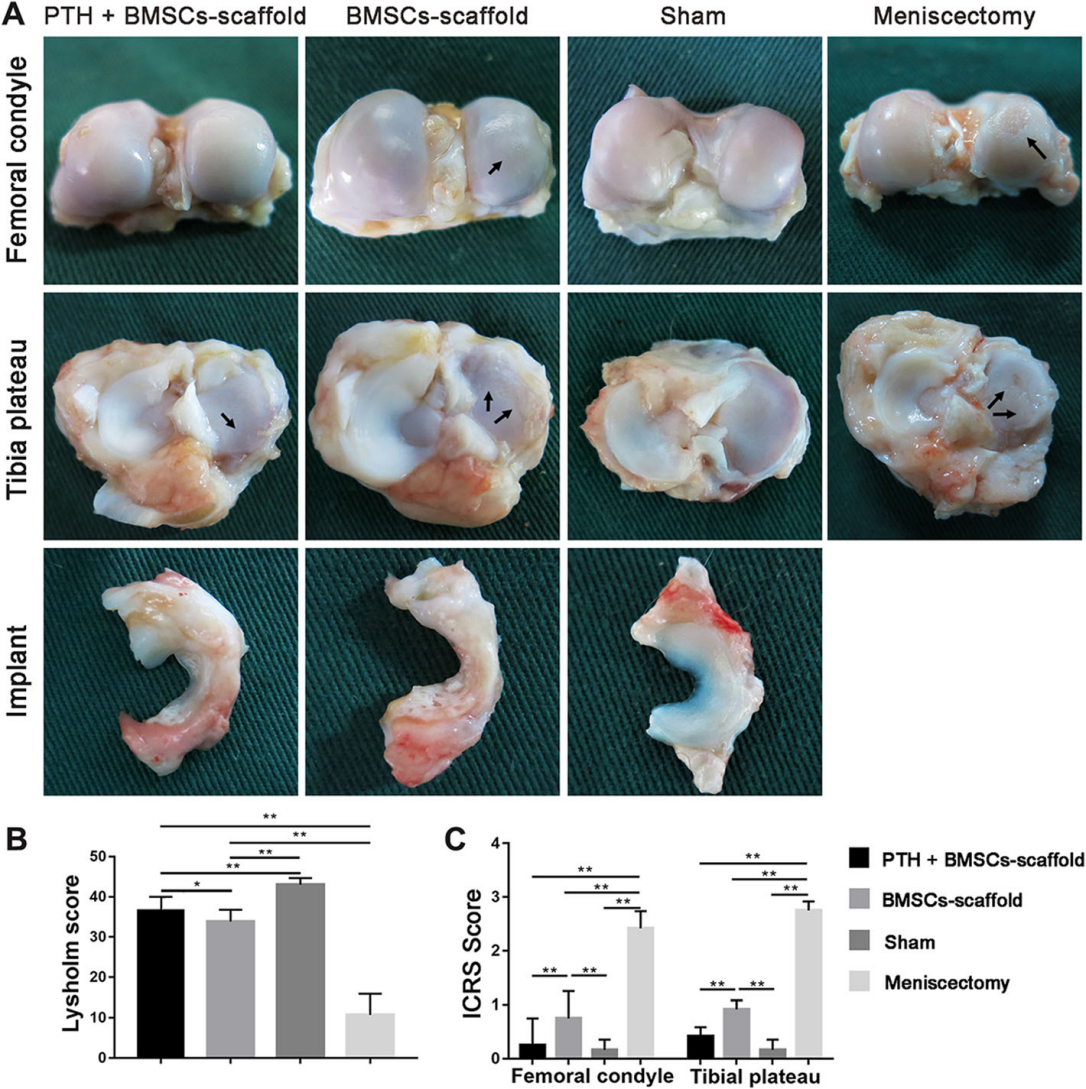
Anatomic observation and scores of the knee joint. **a** Anatomic observation of knee cartilage and meniscus implant (arrows indicate the cartilage injury). **b** Lysholm score, the PTH + BMSCs–scaffold group presented better knee joint function than the BMSCs–scaffold group. **c** ICRS score, the cartilage defect degree of the femoral condyle and tibial plateau extent in the PTH + BMSCs–scaffold group was less than that in the BMSCs–scaffold group and showed no significant difference with that in the Sham group (*P < 0.05, **P < 0.01)

All animals recovered well without infection. Twelve weeks postoperatively, dogs in the Meniscectomy group were capable of walking but found running difficult; those in the BMSCs–scaffold group demonstrated normal walking and running, despite a slight limp in two dogs; whereas those in the PTH + BMSCs–scaffold group exhibited normal walking, running, and climbing of stairs, which was the same as that observed in the Sham group. Lysholm scores for knee joint function (Fig. 3b) of the PTH + BMSCs–scaffold group (36.59 ± 2.04), BMSCs–scaffold group (33.25 ± 2.27), and Sham group (43.00 ± 1.63) were all significantly higher (*P* < 0.01) than that of the Meniscectomy group (10.67 ± 5.21). Further, the score for the PTH + BMSCs–scaffold group was significantly higher than that of the BMSCs–scaffold group (*P* < 0.05).

### Anatomic observations

The anatomic observation (Fig. 3a) found that new tissue formed in all the implants, and most of the scaffolds maintained the original shape and size without fracture or fragmentation. Compared to the BMSCs–scaffold group, the PTH + BMSCs–scaffold group formed more neocartilage-like tissue on the surface of the implant, which was smoother and better integrated with the surrounding tissue.

In the Meniscectomy group, the volume of joint fluid increased in the joint cavity, connective tissue was newly formed, and the joint cartilage surface was severely abraded. For the BMSCs–scaffold group, both the femoral condyle and tibial plateau surface were abraded to some extent. In the PTH-treated group, there was almost no abrasion on the femoral condyle surface and slight abrasion on the tibial plateau surface, which was similar to those in the Sham group. According to the ICRS cartilage lesion classification (Fig. 3c), the score for femur cartilage in the BMSCs–scaffold group (0.92 ± 0.17) was significantly lower than that in the Meniscectomy group (2.42 ± 0.32) (*P* < 0.01), but significantly higher than that in the Sham group (0.17 ± 0.19); while the PTH + BMSCs–scaffold group score (0.42 ± 0.17) was significantly lower than that in the BMSCs–scaffold group (*P* < 0.01) and slightly higher than that in the Sham group with no significant difference. Similar to femoral condylar cartilage, the tibial plateau cartilage defect scores for the PTH + BMSCs–scaffold group (0.42 ± 0.17) and BMSCs–scaffold group (1.24 ± 0.17) were significantly lower than that for the Meniscectomy group (2.75 ± 0.17). The PTH + BMSCs–scaffold group score showed no significant difference as compared to that of the Sham group (0.17 ± 0.19), but was significantly lower than that of the BMSCs–scaffold group (*P* < 0.01).

### Histological evaluation of implants

**Fig. 4:**
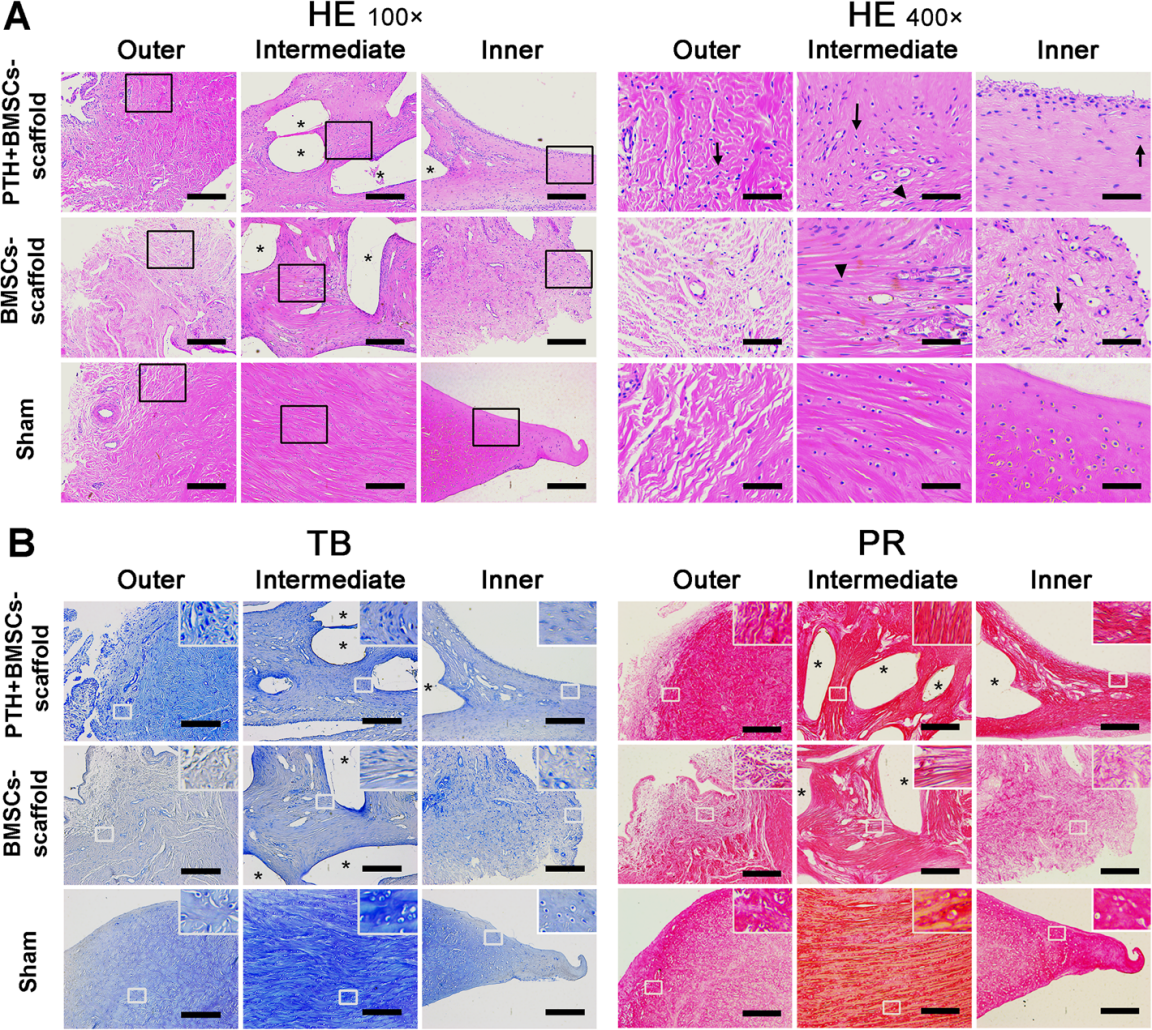
Histological observation of meniscus implants. **a** H&E staining. The general view on the left (scale bar = 100 μm) shows the structure of the outer, intermediate, and inner regions of the new tissue of the meniscus implants, and the high-magnification images on the right (scale bar = 25 μm) correspond to the images inserted in the frame in the left view, respectively. A large number of chondrocyte-like cells (black arrows) were observed in the intermediate and inner regions in PTH + BMSCs–scaffold group implants, which were similar to the native meniscus in the Sham group, while mostly fibroblast-like cells (triangles) were observed in the intermediate region of the implants of the BMSCs–scaffold group. **b** Toluidine blue (TB) and Picrosirius Red (PR) staining showed the deposition of proteoglycan and collagen type I in the new tissue of meniscus implants and native meniscus, respectively. The PTH + BMSCs–scaffold group had a stronger staining than the BMSCs–scaffold group (scale bar = 100 μm). Asterisks marked the dissolution position of the PCL scaffold in the process of sectioning

H&E staining showed that (Fig. 4a) the implanted scaffolds were filled with new tissue; however, there were more voids and connective tissue observed in the new tissue of the BMSCs–scaffold group, while the new tissue in the PTH + BMSCs–scaffold group was more uniform. In the enlarged image of H&E staining (Fig. 4b), the PTH-treated group showed a large number of round-shaped chondrocyte-like cells in the intermediate and inner regions of the implants, which were embedded in lacuna structure, and vascularization and spindle-shaped fibroblast-like cell distribution in the outer regions; these observations were similar to those in the native meniscus of the Sham group. For the BMSCs–scaffold group, there were more spindle-shaped fibroblast-like cells and less chondrocyte-like cells, with no cartilage islands in the intermediate regions. In the case of TB staining and PR staining (Fig. 4b), the PTH-treated group showed stronger staining than the BMSCs–scaffold group. Further, the staining distribution in this group was similar to that seen in the native meniscus, indicating the deposition of more cartilage ECM components proteoglycans and Col1 in the neo-tissue of implants in the PTH + BMSCs–scaffold group.

### Immunohistochemical evaluation of implants

**Fig. 5:**
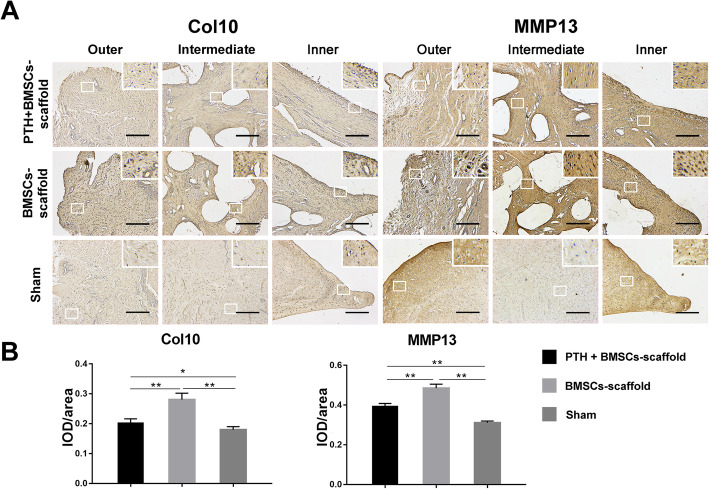
**a** Immunohistochemical staining for Col10 and MMP13 of meniscus implants (scale bar = 100 μm). **b** Immunohistochemical analyses of Col10 and MMP13 of meniscus implants. Values for integrated optical density per area (IOD/area) of Col10 and MMP13 were lower in the PTH + BMSCs–scaffold group compared with those in the BMSCs–scaffold group (*P < 0.05, **P < 0.01)

According to the immunohistochemical staining image (Fig. 5a), terminal differentiation markers Col10 and MMP13 were markedly observed in the cytoplasm in the intermediate and inner regions of the implants in the BMSCs–scaffold group as compared with the Sham group, while they were observed only in the inner region of implants in the PTH + BMSCs–scaffold group. The IOD/area value of immunohistochemistry images (Fig. 5b) supported these observations. The IOD/area value for Col10 in the BMSCs–scaffold group (0.28 ± 0.02) was significantly higher than that in the Sham group (0.18 ± 0.01) (*P* < 0.01) and significantly lower in the PTH + BMSCs–scaffold group (0.20 + 0.01) (*P* < 0.01). The IOD/area values for MMP13 in the PTH + BMSCs–scaffold group (0.39 ± 0.01) and BMSCs–scaffold group (0.48 ± 0.02) were significantly higher than that in the Sham group (0.31 ± 0.01) (*P* < 0.01); however, the value in the PTH + BMSCs–scaffold group was significantly lower than that in the BMSCs–scaffold group (*P* < 0.01).

### Histology and immunohistochemical evaluation of knee articular cartilage

**Fig. 6:**
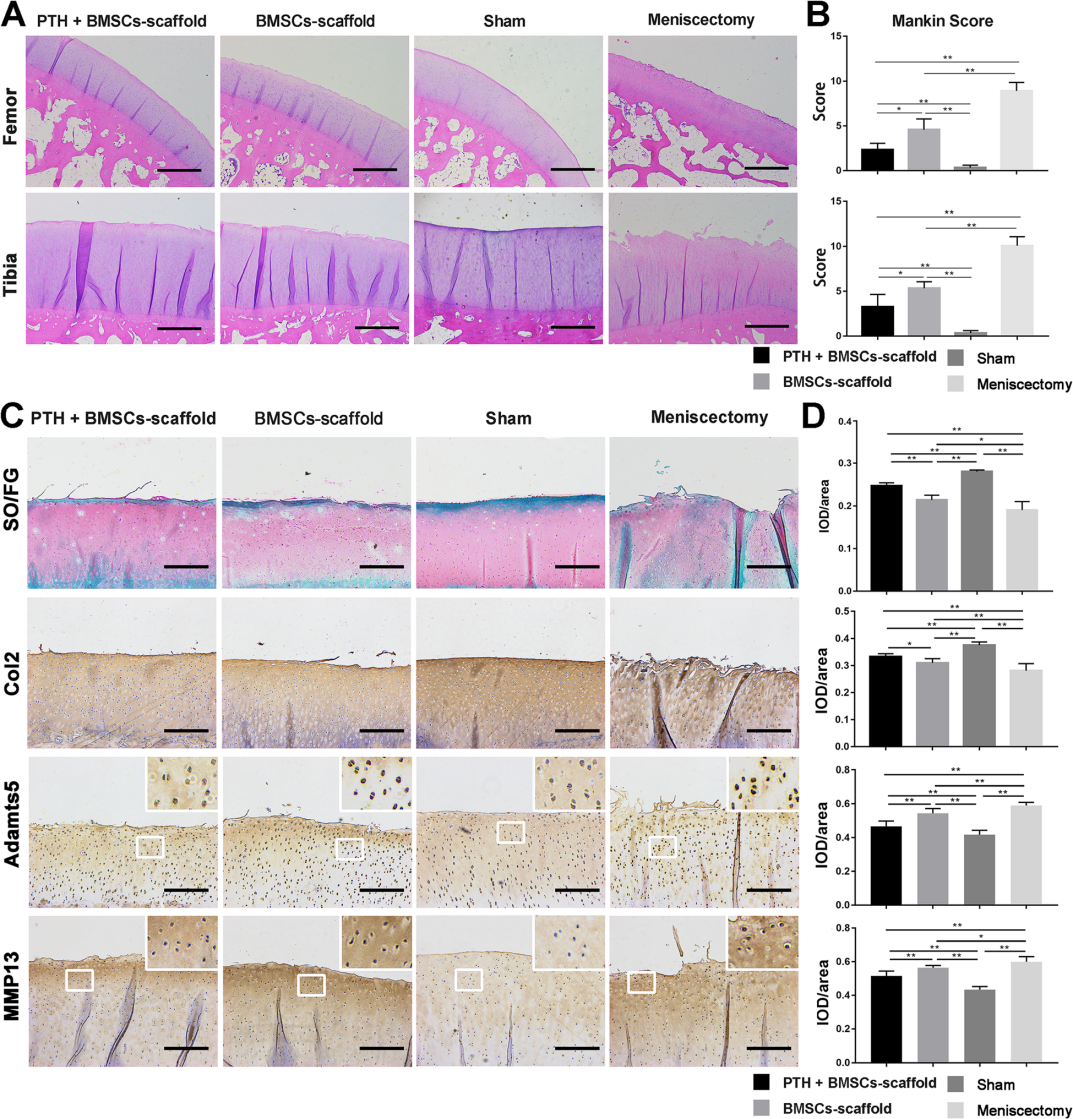
Histological and immunohistochemical observation and evaluation of the knee joint cartilage. **a** H&E staining of the femoral condyle and tibial plateau cartilage (scale bar = 250 μm). **b** The Mankin score of histology of femoral condyle and tibial plateau cartilage. The PTH + BMSCs–scaffold group showed lower cartilage degeneration than the BMSCs–scaffold and the Meniscectomy groups. **c** SO/FG staining and immunohistochemical staining of tibial plateau for cartilage ECM component GAG and Col2 and matrix degradation markers Adamts5 and MMP13 (scale bar = 100 μm). **d** Semiquantitative analysis of SO/FG staining and immunohistochemical staining. Values for integrated optical density per area (IOD/area) of GAG and Col2 in the PTH + BMSCs–scaffold group were higher and those for Col10 and MMP13 were significantly lower than that in the BMSCs–scaffold group (*P < 0.05, **P < 0.01)

In the HE staining (Fig. 6a), both femur and tibia cartilage showed clefts and hypo-cellularity in the Meniscectomy group, and some of the clefts of the tibia cartilage progressed to the radial zone. In the BMSCs–scaffold group, cartilage damage was reduced but still showed irregular clefts to the transitional zone and hypo-cellularity in the superficial zone. In the PTH + BMSCs–scaffold group, the degenerative changes of cartilage were less severe; irregular clefts and hypo-cellularity were observed only in the surface of tibia cartilage, and the femur cartilage was well-preserved. According to the Mankin score (Fig. 6b), the femur cartilage damage degree in the BMSCs–scaffold group (4.50 ± 1.23) was significantly lower than that in the Meniscectomy group (8.92 ± 0.96), but higher than that in the Sham group (0.33 ± 0.27) (*P* < 0.01), and significantly decreased with PTH combination (2.33 ± 0.72) (*P* < 0.05). The Mankin score for the tibia cartilage in the BMSCs–scaffold group (5.33 ± 0.72) was significantly lower than that in the Meniscectomy group (10.08 ± 1.00) and higher than that in the Sham group (0.17 ± 0.19) (*P* < 0.01), and the score in the PTH + BMSCs–scaffold group (3.25 ± 1.40) was significantly lower than that in the BMSCs–scaffold group (*P* < 0.05).

The SO/FG staining and immunohistochemistry analysis (Fig. 6c) found decreased staining of cartilage matrix GAG and Col2 and increased staining of matrix degeneration markers Adamts5 and MMP13 in all the other three groups when compared with the Sham group. The corresponding IOD/area value (Fig. 6d) showed that BMSCs–scaffold (GAG: 0.21 ± 0.01, Col2: 0.31 ± 0.02) significantly impeded the reduction of GAG and Col2 of meniscectomy (GAG: 0.19 ± 0.01, Col2: 0.28 ± 0.03) (*P* < 0.01), and the combination of PTH (GAG: 0.25 ± 0.01, Col2: 0.33 ± 0.01) reserved more. For Adamts5, the IOD/area values were as follows: Meniscectomy group (0.59 ± 0.07) > BMSCs–scaffold group (0.54 ± 0.03) > PTH + BMSCs–scaffold group (0.46 ± 0.03) > Sham group (0.41 ± 0.03), where the significant difference was observed across all four groups (*P* < 0.01). The IOD/area difference of MMP13 was similar to that of Adamts5: Meniscectomy group (0.60 ± 0.03) > BMSCs–scaffold group (0.56 ± 0.01) > PTH + BMSCs–scaffold group (0.52 ± 0.03) > Sham group (0.43 ± 0.19), and the difference was significant across all four groups, respectively (*P* < 0.01).

## Discussion

The purpose of this study was to optimize the 3D printed PCL meniscus scaffolds seeded with BMSCs and cultured in vitro and to investigate the effect of PTH (1-34) on the repairing of the tissue-engineered meniscus in vivo after implantation in the total meniscectomy canine model. The results showed that the BMSCs–scaffold construct synthesized and accumulated more cartilage ECM in vitro when cultured for 21 days as compared to other culture time points. Furthermore, intra-articular injection of PTH (1-34) decreased the cell hypertrophy of the tissue-engineered meniscus during regeneration and increased the chondroprotective effects of the tissue-engineered meniscus for knee cartilage in vivo. This demonstrated the satisfactory efficacy of PTH (1-34) and tissue-engineered meniscus combination on the meniscal replacement.

There are various methods to prepare tissue-engineered meniscus scaffolds. However, 3D printing provides high controllability of the internal structure and geometric shape and personalization of printed entities [5, 27]. Due to the complicated anatomical structure and stress environment of the meniscus, 3D printing has greater advantages in the preparation of tissue-engineered meniscus scaffold as compared to other fabrication technologies [26]. In this study, a PCL scaffold was 3D printed to prepare tissue-engineered meniscus scaffolds, which showed a reproduction of the native meniscus anatomical shape, low degradation rate, and good cell compatibility, and could anatomically respond to the knee joint pressure as well as support the growth of cells and tissues in vivo.

Currently, there is no uniform standard for the in vitro culture of cell-seeded scaffolds in tissue-engineered meniscus studies. In many studies, cell-free scaffold or cell-seeded scaffold was transplanted without in vitro culture into animals; although certain regeneration effects were achieved, joint degeneration often occurred after transplantation [27, 28]. In this study, 3D printed scaffolds were seeded with canine BMSCs and cultured under chondrogenic culture medium in vitro, and the BMSCs–scaffold showed an increasing trend of cell proliferation and ECM, GAG, and Col2 synthesis, which reached the highest levels on day 21 of culture, and declined on day 28; the decline may be related to hypertrophy of BMSCs during long-term chondrogenic induction in vitro. Pelttari et al. [29] found upregulation of hypertrophy-associated genes in MSCs during chondrogenic differentiation in vitro, and extensive calcification of the ECM after ectopic transplantation in mice. Fischer et al. [12] found that MSCs produced PTHrP only during the first 2–3 weeks of chondrogenesis, which was then downregulated and strongly induced Indian hedgehog (IHH) expression, and it is closely associated with matrix mineralization [30]. Therefore, the BMSCs–scaffold construct was cultured in vitro for 21 days before implantation, which makes it more conducive to the synthesis and accumulation of cartilage ECM, resulting in more mature structure and function, so that it responds more quickly to knee joint pressure after implantation. In addition, studies showed that surface modification of the PCL scaffold increases the adhesion, proliferation, and differentiation characteristics of seeded cells, which is an exploration direction for us to further optimize and modify the meniscus scaffold [31, 32].

BMSCs are frequently used as seed cells for tissue-engineered meniscus [10, 33]; however, chondrogenesis leads to undesired terminal differentiation of the generated chondrocyte [34, 35], which reduces the quality of regenerative tissue and decreases its repair efficacy [30]. In our study, the immunohistochemistry of meniscus implants in the BMSCs–scaffold group showed high expression of the chondrocyte terminal differentiation markers Col10 and MMP13, indicating the terminal differentiation of BMSCs. The terminal differentiation program of MSC-derived chondrocytes is similar to that of growth plate chondrocytes, in respect of the expression profiles of many relevant genes [35]. In the growth plate, the maturation and hypertrophic changes of growing cartilage are most prominently regulated by the PTHrP-IHH axis [36, 37]. IHH promotes the proliferation and maturation of chondrocytes; PTHrP inhibits chondrocyte maturation and suppresses the expression of IHH and thereby inhibits chondrocyte hypertrophy [30, 38]; PTHrP thus was considered to be a candidate to inhibit hypertrophy during MSC chondrogenic differentiation; and the effect of PTHrP on inhibiting the hypertrophy of BMSCs chondrogenesis has been reported by a lot of studies [13, 17, 39–41]. In this study, at 12 weeks postoperatively, the cell composition and ECM deposition of the neo-tissue of meniscus implants in the PTH-treated group were similar to those of native meniscus. Furthermore, this tissue also showed lower expression of terminal differentiation markers Col10 and MMP13. These observations suggested that intra-articular injection of PTH (1-34) enhanced tissue regeneration and ECM deposition as well as inhibited the terminal differentiation of tissue-engineered meniscus with BMSCs as seed cells in vivo.

At the same time, we also observed the chondroprotective effect of PTH (1-34) on the tissue-engineered meniscus implant. Although recent studies have attempted to make tissue-engineered meniscus that simulates the anatomy and mechanical properties of the native meniscus to alleviate the stress environment of the knee joint and better protect the knee cartilage, their protective effect after transplantation needs to be strengthened [3, 42, 43]. Hannink et al. [14] implanted PCL-PU meniscus scaffolds in dogs and found articular cartilage degeneration and chondrocyte hypertrophy. Similar to their findings, we also found that the articular cartilage in the tissue of the BMSCs–scaffold group showed significant damage, although it showed a certain reduction in cartilage degeneration and chondrocyte loss when compared with the Meniscectomy group. This may be due to the variation of cartilage friction coefficient in the knee joint after the transplantation, resulting in wear on the cartilage surface; meanwhile, the biomechanical properties of the knee joint may have changed, which would disrupt the normal homeostasis of the joint, leading to cartilage degeneration [43–45]. The degeneration of cartilage may also have an impact on the meniscus function [46].

Adamts5 and MMP13 are considered important catabolic enzymes that degrade aggrecan (AGG) and Col2, key ECM components of functional cartilage, and their expression is related to the cartilage degeneration [19, 47, 48]. The potential of PTH (1-34) in protecting against cartilage degeneration and inducing matrix regeneration after articular cartilage injury has been demonstrated in in vivo studies [19, 40, 49]. Dai et al. subcutaneously injected PTH (1-34) into guinea pigs of the meniscectomy model and found the inhibition of cartilage degeneration by PTH (1-34), which may be related to the inhibition of Adamts4 and MMP13 expression [50]. In this study, the injection of PTH (1-34) after transplantation of tissue-engineered meniscus reduced the degree of lesions in the knee cartilage which showed a higher expression of GAG and Col2 and lower expression of Adamts5 and MMP13. This suggested that PTH (1-34) inhibited the degeneration of cartilage caused by the total substitution of the tissue-engineered meniscus, protected the integrity of the knee joint cartilage, and thus enhanced the repairing effects of the tissue-engineered meniscus.

There are some limitations to this study. Firstly, although PTH (1-34) promoted the repair effect of BMSCs–scaffold in vivo, further investigations of the specific mechanisms need to be performed, such as receptor mediation and intra-cellular signal pathway of PTH (1-34) in BMSCs and articular chondrocytes and the changes of tissue and protein expression in different time point after transplantation of scaffold. Secondly, although our data indicated that PTH (1-34) functions through reducing hypertrophy of BMSCs and maintaining articular cartilage ECM metabolism, other mechanisms that may play a role also need to be further investigated, for example, whether PTH functions through affecting paracrine effects of MSCs. Thirdly, the injection of PTH (1-34) was carried out only with a single dosage and a single time windows; although it promoted the regeneration effects of BMSCs–scaffold, cartilage degradation was not completely prevented. Studies reported that the response to PTH (1-34) treatment varies with dosage and timing [21, 51–53]; therefore, different dosage gradients and time windows need to be established in our future study to explore more effective schemes of PTH (1-34) administration.

## Conclusions

In this study, we found that 21 days is the optimal time for in vitro culturing of the tissue-engineered meniscus based on the 3D-printed PCL scaffold seeded with canine BMSCs. Further, PTH (1-34) application promoted the regenerative and chondroprotective effects of the tissue-engineered meniscus total implantation in a canine model by inhibiting the terminal differentiation of BMSC chondrogenesis and degeneration of knee joint cartilage. Thus, this therapeutic combination represents a promising method to increase the chance of regeneration in the tissue-engineered meniscus.

## Data Availability

All data have been included in this article and its supplementary information files.

## Abbreviations

BMSCs: Bone marrow mesenchymal stem cells
3D: Three-dimensional
PTH: Parathyroid hormone
PTHrP: Parathyroid hormone-related protein
PCL: Poly(e-caprolactone)
DMEM: Dulbecco’s modified Eagle’s medium
GAG: Glycosaminoglycan
ECM: Extracellular matrix
OD value: Optical density value
ICRS: International Cartilage Repair Society
H&E staining: Hematoxylin and eosin staining
TB staining: Toluidine blue staining
PR staining: Picrosirius red staining
SO/FG staining: Safranin-O/Fast Green staining
COL: Collagen
IHH: Indian hedgehog
MMP13: Metalloproteinases-13
Adamts5: A disintegrin and metalloproteinase with thrombospondin motifs 5
